# Visualization of Molluscum Contagiosum Virus in FFPE Skin Sections Using NanoSuit‐CLEM: Ultrastructural Evidence of Viral Spread via Skin Barrier Disruption

**DOI:** 10.1002/iid3.70212

**Published:** 2025-06-03

**Authors:** Yuri Sakano, Hideya Kawasaki

**Affiliations:** ^1^ NanoSuit Research Laboratory, Division of Preeminent Bioimaging Research, Institute of Photonics Medicine Hamamatsu University School of Medicine Hamamatsu Shizuoka Japan

**Keywords:** Molluscum contagiosum, NanoSuit‐CLEM, skin, viral particles

## Abstract

**Background:**

Molluscum contagiosum (MC) is a common viral skin infection caused by members of the Poxviridae family. It primarily affects children, sexually active adults, and immunocompromised individuals. Although MC spreads through direct contact and auto‐inoculation, the precise mechanisms by which the virus penetrates the skin barrier remain poorly understood.

**Methods:**

We applied NanoSuit‐correlative light and electron microscopy (NanoSuit‐CLEM) to formalin‐fixed paraffin‐embedded (FFPE) skin sections to visualize MC virus particles in situ with high resolution. Melan‐A immunohistochemistry using 3,3′‐diaminobenzidine (DAB) with osmium staining was performed to identify Henderson–Patterson bodies.

**Results:**

Ultrastructural analysis revealed that MC virus particles were densely localized in the stratum corneum but did not invade deeper epithelial layers in intact skin. However, in areas of epidermal disruption, such as detached or damaged stratum corneum, the virus was observed penetrating into lower layers. While Melan‐A immunostaining successfully detected Henderson–Patterson bodies, it failed to identify mature MC virus particles. In contrast, NanoSuit‐CLEM combined with Mayer's hematoxylin and lead staining enabled detailed visualization of mature viral particles and their distribution within the stratum corneum.

**Conclusions:**

These findings provide direct ultrastructural evidence that MC virus entry occurs through compromised skin, underscoring the crucial role of the stratum corneum in barrier function. This study highlights the importance of preventing mechanical skin injury, such as scratching or shaving, to limit MC transmission. NanoSuit‐CLEM offers a powerful new tool for studying viral pathogenesis in archival tissue samples.

## Graphical abstract

Molluscum contagiosum (MC) virus, a member of the Poxviridae family, primarily spreads through skin contact, with disrupted skin serving as a key entry point. Using NanoSuit‐correlative light and electron microscopy (CLEM), we visualized MC virus particles densely packed in the stratum corneum but not penetrating intact epithelial layers. This study highlights the role of skin injury in MC transmission and provides a novel method for precise viral mapping, contributing to improved prevention strategies.

## 1 Introduction

*Molluscum contagiosum* virus (MCV) is an unclassified member of the family *Poxviridae* that encompasses viruses of types I–IV [1]. *Molluscum contagiosum* (MC) is endemic in densely populated communities and areas with poor hygiene and health [2], where it primarily affects children, sexually active adults, and immunodeficient individuals [3, 4]. Viral skin infections, including those caused by MCV, are frequently observed in primary care and dermatology practices worldwide.

Although MCV infection is usually self‐limiting in immunocompetent individuals, it may become persistent or disseminated in those with impaired immune function, such as children, patients with atopic dermatitis, and individuals with HIV infection. T cell–mediated adaptive immune responses are essential for the clearance of MCV, suggesting a pivotal innate and adaptive role for immunity in controlling the virus. Individuals with compromised cellular immunity often exhibit more severe or prolonged infections, indicating that CD4+ and CD8 + T cells contribute to the resolution of lesions [5]. However, MCV encodes immune evasion proteins that inhibit the cytokine response, further highlighting the complexity of the virus–host interaction [6].

MCV can spread within an individual via autoinoculation by touching or scratching lesions. The stratum corneum, which serves as a mechanical and immunological barrier, is the first line of defense against viral invasion [7]. Disruption of this barrier via trauma, scratching, or inflammatory skin diseases is known to facilitate viral entry; however, the precise mechanism by which this occurs remains unclear. Conflicting reports have rendered the infection process, with an initial spread by simple contact with seemingly intact lesions [8, 9] or damage to lesions the subsequent transfer of core virus material [10] suggested modes of infection, and it remains uncertain whether acquired adaptive immunological defense is the major defense mechanism against MCV [5].

The direct observation of viral particles may help elucidate the mechanism by which MC spreads. Melan‐A (also known as MART‐1) is a melanocytic marker that was originally developed for melanoma diagnosis but has been reported to cross‐react with the Henderson–Patterson bodies found in MC lesions. Previous studies have demonstrated that Melan‐A staining can supplement H&E staining to aid diagnosis in cases where inflammation or limited biopsy material obscures the typical viral morphology. However, previous studies have also suggested that its use may be limited to highlighting cytoplasmic inclusions such as Henderson–Patterson bodies, and that it may not identify mature virus particles [11, 12]. Clarifying this distinction is important for defining the diagnostic utility of Melan‐A and interpreting immunohistochemical findings in the context of MCV infection.

MCV replicates in the cytoplasm of keratinocytes, where it forms large eosinophilic inclusion molluscum (Henderson–Patterson) bodies. Viral replication occurs in the suprabasal layers of the epidermis, and mature virus particles are released onto the skin surface via keratinocyte lysis or degeneration [5]. These processes contribute to the characteristic central crater of MC lesions. However, most studies investigating MCV replication rely on conventional electron microscopy such as TEM, or histology techniques that utilize light microscopy [5]; thus, ultrastructural insights, especially in terms of the FFPE sections correlated with H&E staining, remain limited. We previously demonstrated that the NanoSuit method offers a non‐destructive method for the high‐resolution 3‐dimensional (3D) observation of wet specimens [13, 14, 15], and the current study provides ultrastructural evidence of viral localization in relation to skin barrier integrity, offering new insights into how MCV bypasses the host defenses in the initial infection process.

In this study, light microscopy and field emission scanning electron microscopy (FE‐SEM) were performed to observe MC virus particles in formalin‐fixed paraffin‐embedded (FFPE) sections in a using correlative light and electron microscopy (CLEM) in a non‐destructive manner under the NanoSuit method. The findings are particularly relevant to readers interested in cutaneous immunity, viral pathogenesis, and inflammation‐related skin disorders.　As cutaneous viral infections often involve complex interactions between barrier integrity and immune surveillance, our findings offer a novel ultrastructural perspective that is directly relevant to clinical and immunological dermatology.

## 2 Materials and Methods

### 2.1 Preparation of Clinical Samples

Paraffin sections were prepared from skin tissues with MC. Approval for the study was obtained from the Research Ethics Boards of Hamamatsu University School of Medicine (approval number 18‐139) and the Chutoen General Medical Center (No. 147). Informed consent was obtained from all participants. All data were anonymized and privacy measures rigorously implemented to protect the personal information of participants, ensuring that the data were used solely for research purposes.

### 2.2 Sample Preparation

FFPE sections were stained with hematoxylin for 20 min and eosin for 10 min then imaged using a NanoZoomer‐RS digital camera (Hamamatsu Photonics Co. Hamamatsu, Japan) or other digital cameras, as described previously [8]. Immunohistochemical (IHC) staining of the paraffin sections was performed using mouse monoclonal anti‐MART‐1/Melan‐A antibody (A103; Roche Diagnostics K. K. Tokyo, Japan) for MC over 30 min at room temperature (20°C–28°C) and visualized with 3,3′‐diaminobenzidine (DAB) for 10 min.

### 2.3 Light and Electron Microscopy Observation of Paraffin Sections Under Application of the Nanosuit Method

For observation, an optimal amount of NanoSuit solution (Solution II; NanoSuit Inc. Hamamatsu, Japan) was added to the glass slides to cover the entire specimen [13, 14, 16] and left for 30 s. Any excess solution was then removed and a thin uniform film produced using a Slide Spinner (Labnet International Inc. NJ, USA; 4800 rpm, 10 s) to ensure optimal imaging under vacuum conditions. NanoSuit Solution II is a proprietary polymer‐based compound that is optimized for the preservation of hydrated biological specimens under vacuum conditions. Although the exact composition has not been disclosed by the manufacturer, the solution was applied in accordance with the supplier's recommended protocol. To observe DNA‐containing MC virus particles under FE‐SEM, DAB‐stained slides were incubated with a 1% osmium solution for 5 min at room temperature and washed with distilled water three times before application of the modified Sasaki's method [17]. FFPE slides were stained with Mayer's hematoxylin (MUTO PURE CHEMICALS CO., LTD, Tokyo, Japan) for 5 min at room temperature and rinsed under running tap water for 5 min to achieve proper bluing before gently dipping in distilled water. Sections were then covered with undiluted lead stain solution for 1 min at room temperature (Merck KGaA, Darmstadt, Germany) and briefly rinsed in distilled water for FE‐SEM observation. FE‐SEM was performed with a Hitachi S‐4800 instrument operated at an acceleration voltage of 5.0 kV in backscattered electron (BSE) mode, as described previously [13].

## 3 Results

### 3.1 Observation of MC Virus Particles Using the NanoSuit‐CLEM Method

**Figure 1:**
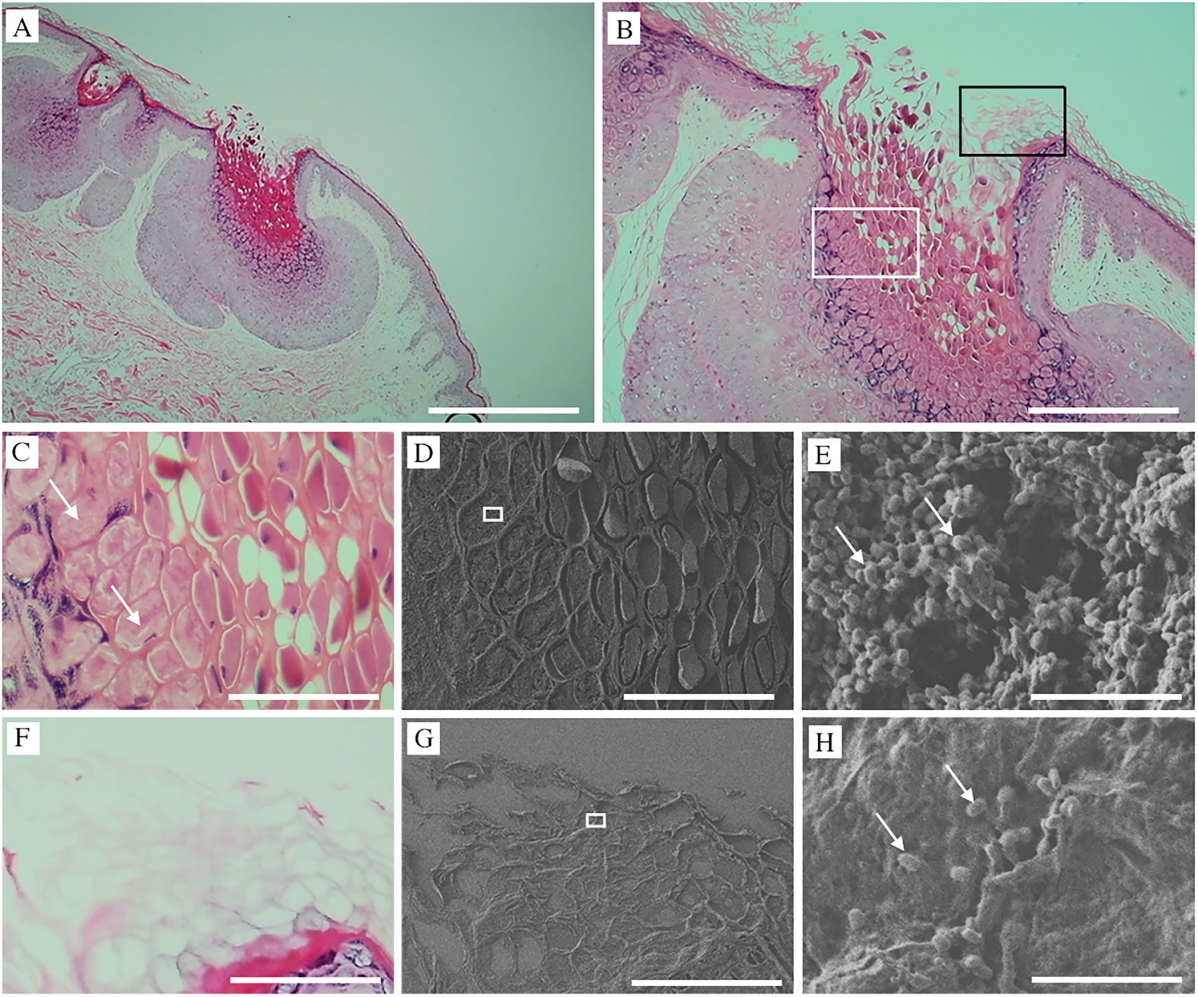
CLEM image of *Molluscum contagiosum* (MC). (A) Cup‐shaped lesion with inverted hyperplastic squamous epithelium lobules extending into the underlying dermis. H&E image. Bar represents 1 mm. (B) Magnified image of (A). Bar represents 0.4 mm. (C) Magnified image of white square in (B). Arrow indicates the eosinophilic Henderson–Patterson bodies (molluscum bodies). Bar represents 120 μm. (D) CLEM image from field FE‐SEM corresponding to (C). Bar represents 120 μm. (E) Magnified image of white square in (D). Arrows indicate MC particles. Bar represents 6.7 μm. (F) Magnified image of black square in (B). Bar represents 120 μm. (G) CLEM image from field FE‐SEM corresponding to (F). Bar represents 120 μm. (H) Magnified image of white square in (G). Arrows indicate MC particles. Bar represents 6.7 μm.

Histopathological examination of MC revealed a cup‐shaped lesion with inverted squamous hyperplastic epithelium lobules extending into the underlying dermis. These intraepidermal lobules are partitioned by connective tissue septa with molluscum bodies (MBs) appearing as round or oval cells undergoing keratohyalin degeneration. The crateriform openings of the MC lesions discharge degenerated cells and keratin onto the skin surface (Figure 1A,B). The coalescence of fine granules within the cytoplasm into eosinophilic Henderson–Patterson bodies can be seen in Figure 1C, which is a in magnification of the white square in Figure 1B (MBs; Figure 1C, arrows). Figure 1D is a CLEM image from a field FE‐SEM corresponding to Figure 1C, in which the magnified square area shows numerous MC virus particles (200–300 nm in diameter; Figure 1E, arrows). The black square in Figure 1B is magnified in Figure 1F, where a keratinized area is observed. The magnified square area in Figure 1G shows multiple MC virus particles (200–300 nm in diameter; Figure 1H, arrows). These results demonstrate the successful use of the NanoSuit‐CLEM method to visualize microstructures such as MC particles, which are undetectable under light microscopy.

### 3.2 Melan‐A Detects MC Infection But Not Mature MC Virus Particles

**Figure 2:**
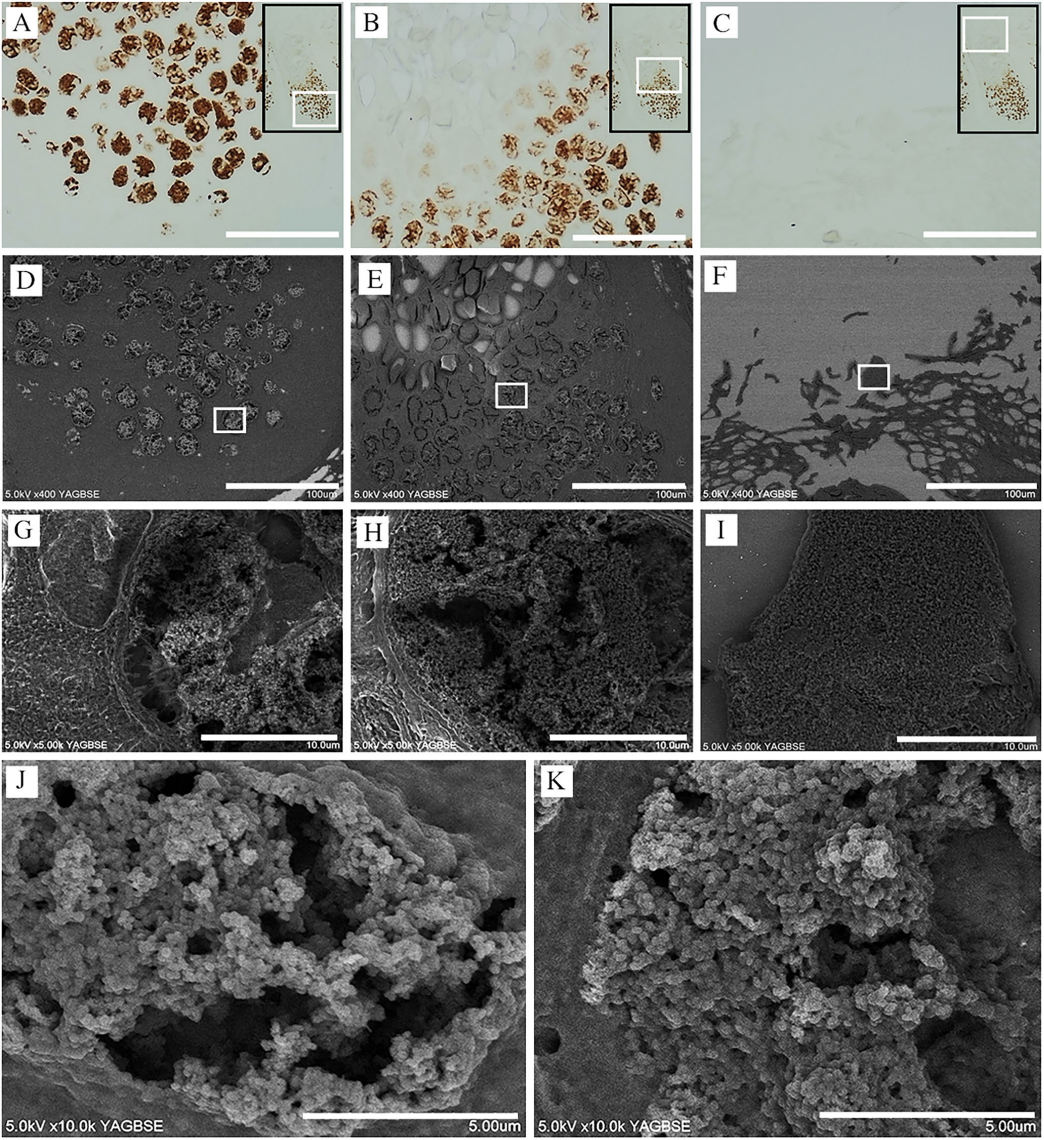
Immunohistochemical analysis of Melan‐A in *molluscum contagiosum* (MC) bodies. (A–C) The MC specimen is immune‐stained with Melan‐A antibody. Black square indicates the entire infected area, and the white square within the black square is the area in which each figure is magnified. (A) Lower part of the MC‐infected area. Melan‐A is positive for Henderson–Patterson bodies. (B) Middle part of the MC‐infected area. (C) Upper part of the MC infected area. White bar represents 100 μm. (D–F) Corresponding CLEM SEM images of A–C, respectively. DAB‐positive, with Melan‐A stained areas depicted as white areas in the SEM‐BSE images. White bar represents 100 μm. (G–I) Magnified images of D–F (white square), respectively. (G and H) A mosaic and mixed pattern of Melan‐A positive/negative MC viral particles are observed. Melan‐A expression weakens MC viral particles in the higher layers of the skin. White bar represents 10 µm. (I) Melan‐A is scarcely expressed in the upper part of the MC‐infected area. White bar represents 10 µm. (J) Magnified image from the lower part of the lesion. White bar represents 5 µm. (K) Magnified image of the middle part of the MC‐infected lesion. White bar represents 5 µm.

MC‐induced infectious lesions are generally identified using immunohistochemistry [14]. Melan‐A is very sensitive for detecting MC bodies [11, 12] and has been found positive for Henderson–Patterson bodies (Figure 2A,B), with weaker effect in the upper layers (Figure 2B,C). Figure 2D–F show the corresponding CLEM‐SEM images for Figure 2A–C, respectively, with the DAB‐positive Melan‐A‐stained areas depicted as the white areas in the SEM‐BSE images. Figure 2G–I are magnified images of Figure 2D–F (white squares), respectively, and show a mosaic and mixed pattern of Melan‐A‐positive/negative MC viral particles (Figure 2G,H). Figure 2J,K show magnified and clearer images from the lower and middle parts of the lesion, respectively. These results suggest that Melan‐A cannot be used to observe mature MC particles.

### 3.3 Observation of MC Virus Spreading Pattern in FFPE Sections Using the Nanosuit Method

**Figure 3:**
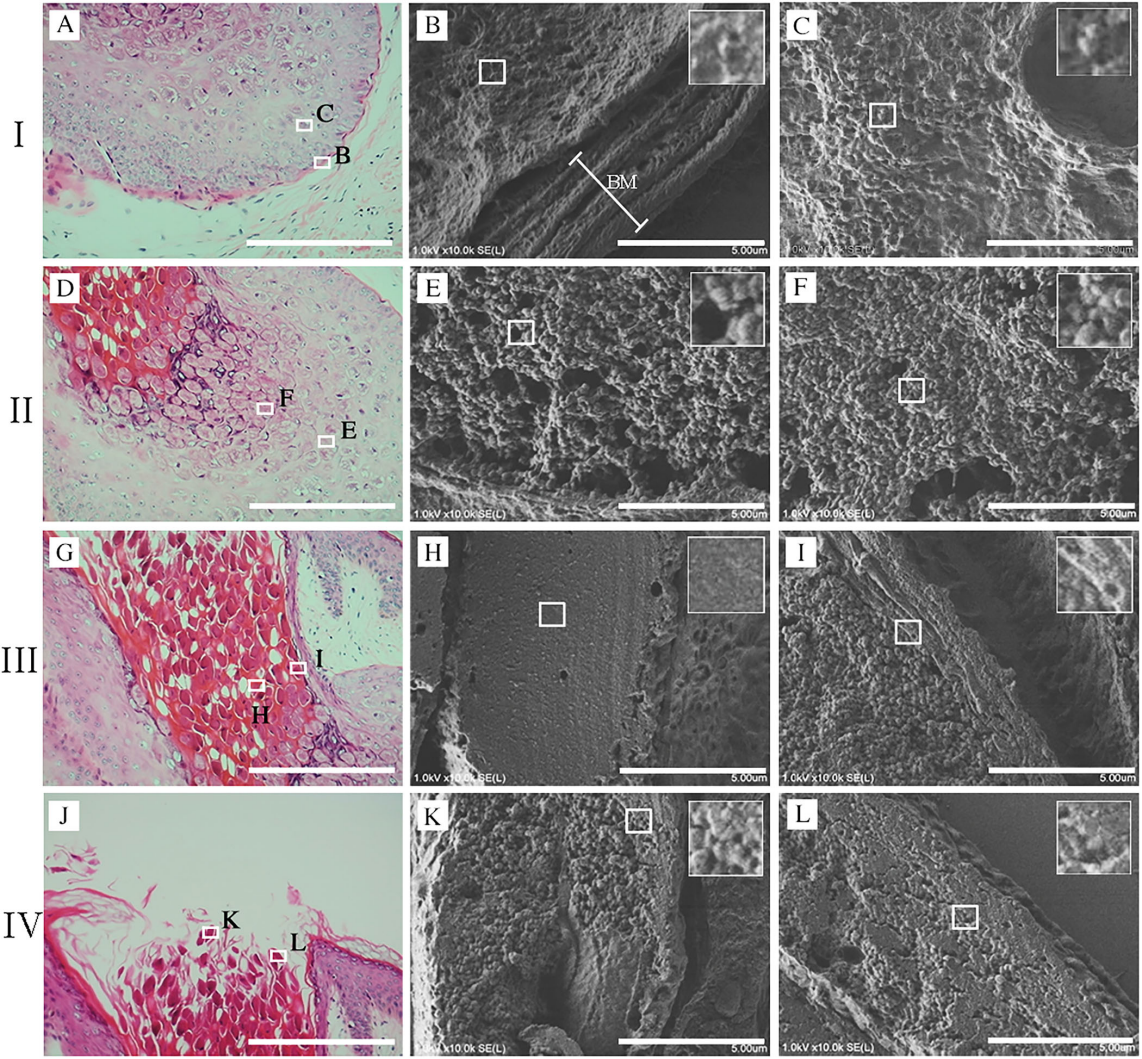
Identification and distribution of MC virus particles in FFPE sections using NanoSuit‐CLEM observation. (A) Overview of the analyzed lesion, divided into four parts (I–IV) for examining the virus particle distribution. Portions B and C (white squares) are selected from part I, representing different regions within the lesion. White bar represents 40 μm. (B) Magnified view of Portion B, adjacent to the basal membrane (BM; stratum basale). A few virus‐like particles are visible in the cytoplasm; however, none cross or are located outside the BM. (C) Magnified view of Portion C, located in the bottom layer of the stratum spinosum. A greater number of MC viral particles are observed in the cytoplasm compared to the BM. White bar represents 5 μm. (D) Part II represents the stratum spinosum, containing multiple MB with eosinophilic cytoplasmic inclusions. White bar represents 40 μm. (E) The lower layer of the stratum spinosum shows numerous virus particles within MB. White bar represents 5 μm. (F) The upper layer of the stratum spinosum displays a higher density and greater number of virus particles in MB. Insets in (E and F) show magnified views of the white squares. (G–L) Parts III and IV represent the stratum corneum, where the lesion center disintegrates, forming a central crater. (G) H&E‐stained section of part III. The white bar represents 40 μm. (H) Virus particles in the central region appear densely packed and indistinguishable, forming a rock‐like flat surface. The white bar represents 5 μm. (I) In another region of the stratum corneum, virus particles remain distinguishable but are densely packed. White bar represents 5 μm. (J) H&E‐stained section in part IV.　White bar represents 40 μm. (K, L) As virus aggregates move upward through the stratum corneum, the solid mass gradually loosens and virus particles become increasingly distinguishable. White bar represents 5 μm.

NanoSuit‐CLEM observation is appropriate for identifying single MC virus particles and understanding their spreading patterns in FFPE sections. To observe and analyze the spread of virus particles, an MC‐infected lesion was divided into four parts (I–IV). Portions B and C (white squares) were selected from part I (Figure 3A). Portion B represents the area adjacent to the basal membrane (BM) (stratum basale). A few virus‐like particles were observed in the cell cytoplasm; however, none were observed to have crossed the BM. The inset shows the white squares magnified at 3x (Figure 3B). Square C in Figure 3A corresponds to the bottom layer of the stratum spinosum, where a greater number of MC viral particles were observed in the cell cytoplasm as compared to the stratum basale (Figure 3C). Part II represents the stratum spinosum layer containing multiple MBs. MBs comprise large suprabasilar epidermal cells with (granular) eosinophilic cytoplasmic inclusions filling the cytoplasm and a small peripheral nucleus. The lower layer of the spinosum in the MB contained numerous virus particles (Figure 3E), whereas the upper layer showed a higher density and greater numbers of virus particles (Figure 3F). Magnified views of the white squares can be seen in the insets of Figure E and F, with parts III and IV representing the stratum corneum layer (Figure 3G–L). The stratum corneum ultimately disintegrates at the center of the lesion, releasing MBs together with keratinous debris to form a central crater in which the virus particles are packed and hardly distinguishable, with a rock‐like flat surface appearance, as seen in Figure 3H. The multiple virus particles in other parts of the stratum corneum are packed but still distinguishable. The viral particles do not appear to have invaded the epithelial surface (Figure 3I). Notably, the viral aggregate, which is in the form of a solid mass in the lower layer of the stratum corneum, loosens as it moves upward, with the viral particles gradually becoming distinguishable (Figure 3J–L).

### 3.4 Investigation Into the Invasion of the MC Virus Into the Epithelium in the FFPE Sections Using the Nanosuit Method

**Figure 4:**
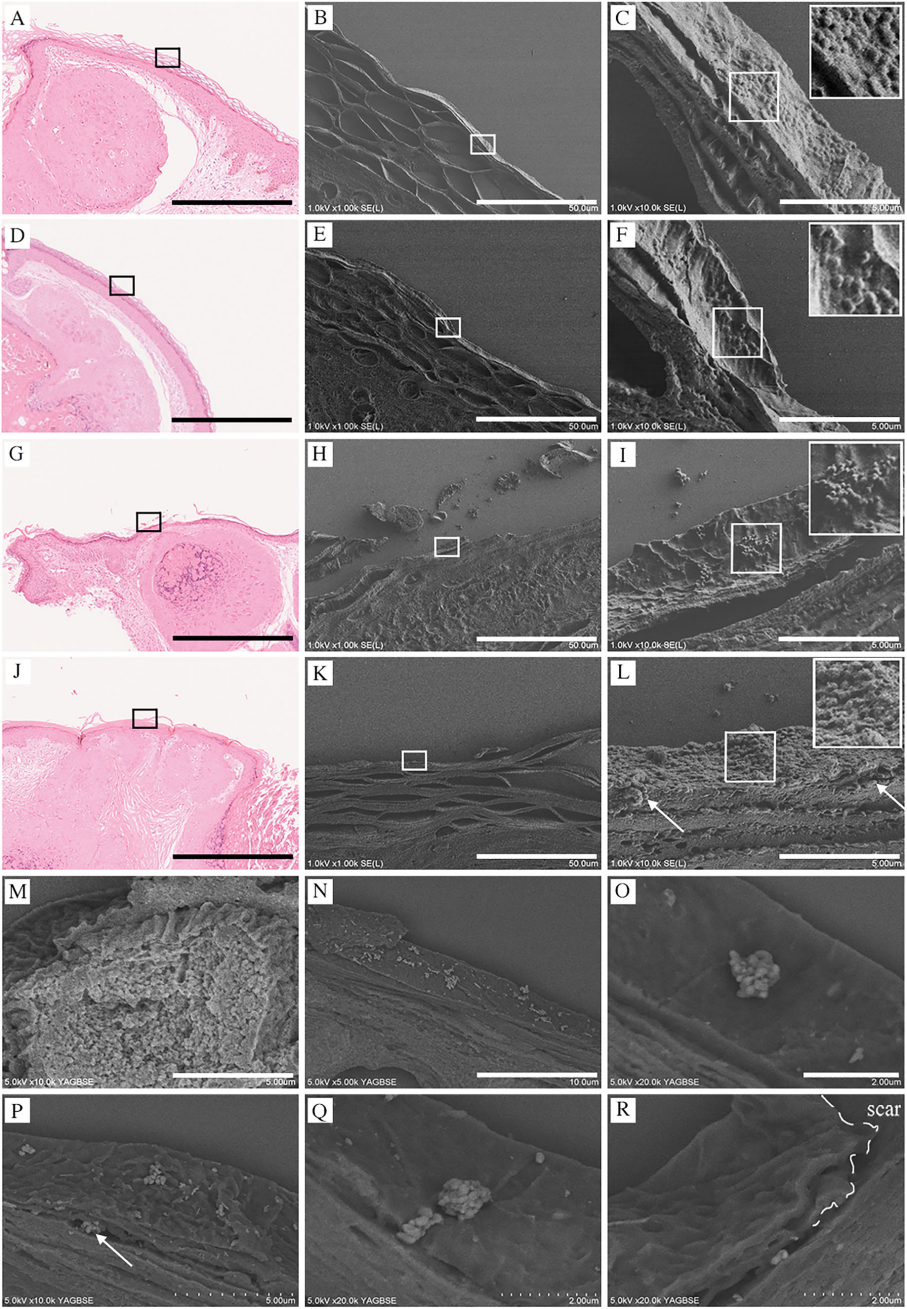
Analysis of MC virus invasiveness to the epithelium using the NanoSuit‐CLEM method. (A–C) Case 1: Multiple MC virus particles attached to the stratum corneum surface. Most virus particles exhibit a dome‐shaped deformation, indicating firm attachment to the surface. (D–F) Case 2: Similar findings with dome‐shaped MC virus particles attached to the surface. (G–I) Case 3: Some virus particles retain a spherical shape, suggesting weaker attachment. A few particles are observed beneath the epithelial layer (arrows), indicating potential viral infiltration. (J–L) Case 4: Further evidence of dome‐shaped virus particles attached to the corneum surface, showing minimal penetration into deeper layers. (A, D, G, J) H&E staining section. Black bar represents 600 μm. (B, E, H, K) FE‐SEM image (BSE mode). Magnification of black squares of A, D, G, J. Black bar represents 50 μm. (C, F, I, L) FE‐SEM image (BSE mode). Magnification of black squares in B, E, H, K. Black bar represents 5 μm. (M–R) FE‐SEM images following Mayer's hematoxylin and lead staining. (M) Virus aggregation in the Melan‐A‐negative upper layers depicted as white‐stained areas in BSE mode. White bar represents 5 μm. (N, O) Most viral particles remain on the corneum surface. (N) White bar represents 10 μm. (O) White bar represents 2 μm. (P) A few virus particles are observed beneath the corneum (arrow). White bar represents 5 μm. (Q) Virus particles appear lodged in a groove, with some infiltration into deeper areas (arrow). White bar represents 2 μm. (R) Viral infiltration is observed in damaged areas of the corneum (dashed line, arrow). White bar represents 2 μm.

The epithelial invasiveness of MC virus particles in different clinical cases was analyzed using CLEM (Figure 4). Four representative cases are presented to observe commonalities and their variations: 1 (Figure 4A–C), 2 (Figure 4D–F), 3 (Figure 4G–I), and 4 (Figure 4J–L). No signs of erosion were observed where the epithelial structure remained intact, allowing assessment of the unaltered epithelium. Multiple MC virus particles were attached to the corneum surface, with most particles forming a dome‐like shape, suggesting firm attachment (Figure 4C,F,L), and some retaining their spherical shape, indicating weaker attachment (Figure 4I). Most MC virus particles did not penetrate or invade the deeper layers; however, a few were observed beneath the epithelium (Figure 4I, arrows).

Previous findings have indicated that enhancing viral particles using IHC methods is challenging due to a lack of antibodies against the MC virus surface markers that are suitable for use with FFPE sections (Figure 2). To address this issue, an alternative method to enhance the visualization of the viral particles was applied in which aggregated viruses in the Melan‐A‐negative upper layers are depicted as a white‐stained area in FE‐SEM under the BSE mode (Figure 4M). Notably, each MC particle was clearly distinguishable (Supplemental Figure 1A,B), with most viral particles observed on the corneum surface (Figure 4N,O) and some beneath the corneal layer (Figure 4P, arrow). A cluster of viral particles was observed to be lodged in the groove, with images suggesting that the virus was infiltrating from the deeper groove area (Figure 4Q, arrow). The images also suggested that the MC virus entered through areas in which the corneum was damaged (dashed line), infiltrating via the detached portions (Figure 4R, arrow).

## 4 Discussion

This report presents the first case of observing MC‐infected skin tissue in which SEM is used to observed a section identified by H&E staining. Previous studies have observed the viral replication mode using transmission electron microscopy (TEM); however, these observations were limited to localized and 2D views. In contrast, the method used in this study is a revolutionary approach by which the replication mode of individual 3D virus particles at different sites (I–IV) can be observed while capturing the overall structure. The NanoSuit method allows for non‐destructive sample processing, enabling repeated observations with SEM and optical microscopy [13, 14, 16], and enables pathologists to observe regions of interest that cannot be detected by optical microscopy at ultrahigh magnification, significantly enhancing lesion observation. Previous studies have shown that MCV replication occurs in suprabasal keratinocytes, with virus particles accumulating within the cytoplasmic inclusion bodies before release via cellular degeneration [5, 18]. Our observation of the increasing viral particle density from the stratum basale to the upper spinosum and stratum corneum aligns with this model. In particular, the lack of penetration through the intact basal membrane supports the notion that MCV is strictly epidermotropic with no dermal invasiveness, underscoring the importance of epithelial integrity for viral containment. The aggregation and overlap of viral particles, especially in the stratum corneum, and the limitations of FFPE‐derived contrast variation, meant that the use of automated quantification with image analysis software was not feasible. Instead, we relied on representative image comparisons across layers, which revealed consistent qualitative trends in terms of the viral distribution. Future studies using immunogold labeling or serial‐section 3D reconstruction may enable more precise particle‐level quantification.

Melan‐A is a good marker for detecting MC bodies [11, 12]. However, our results showed that it could not identify mature MC virus particles, and the target antigen of the MC virus that Melan‐A recognizes is yet to be identified. These results lead to the conclusion that Melan A cross‐reacts with early viral cytoplasmic inclusions (Henderson–Patterson bodies), likely due to structural similarity or epitope exposure in the immature stage of virus assembly. Melan‐A‐recognized antigen loss occurs as the MC virus matures, owing to Melan‐A‐recognized specific proteins cleavage via proteases and other enzymes, diminishing certain protein components. Proteins detected in the early stages may therefore not be present in the mature stages. Structural changes in a virus can obscure its epitopes, rendering antibody detection difficult, and certain proteins may be degraded after fulfilling their roles, rendering them no longer detectable in mature viruses. Currently, no antibodies validated for the detection of mature MCV particles in FFPE tissues are commercially available, and a polyclonal antibody targeting MCV that was previously described by Penneys et al. (1986) is no longer widely used [19], underscoring the need for the development of new immunohistochemical reagents that can reliably detect the various stages of MCV in clinical specimens. The development of new antibodies that can detect mature MC virus particles in FFPE sections is thus warranted in future studies. The mature MCV virion comprises several structural proteins (MC049L, MC113L, etc.) and envelope‐associated proteins (MC142R, MC146R, etc.), which have immunomodulatory functions [20]. Among these, one has been characterized and may serve as a promising candidate for future antibody development. The development of antibodies against these mature virion‐associated proteins is likely to enhance the diagnostic sensitivity and allow for the stage‐specific immunohistochemical detection of MCV infection. Such advancements would improve the correlation between viral morphogenesis and tissue pathology, particularly in cases with significant inflammation or in immunocompromised patients. Therefore, further proteomic studies and targeted antibody development are required to establish reliable markers for mature MCV particles in routine histopathological specimens.

The alternative method that uses meyer‐lead staining was invented as an easy‐to‐use staining method for staining ultra‐thin sections for TEM instead of uranyl acetate [17]. To the best of our knowledge, this is the first study in which the proposed method was applied to FFPE sections for SEM, and the results showed clear identification of MC virus particles on the corneum surface. These ultrastructural observations reinforce the clinical understanding that intact skin acts as an effective barrier to MCV infection and support models in which mechanical disruption of the stratum corneum, via scratching, eczema, or minor trauma, facilitates viral entry. This is consistent with the clinical observation that individuals with atopic dermatitis or other chronic skin conditions exhibit more widespread and persistent MC lesions. Furthermore, the implications for pediatric and immunocompromised populations are significant. Given the increased vulnerability of these groups, promoting skin integrity and avoiding behaviors that damage the epidermis (e.g., scratching, shaving) should be considered a cornerstone of MC prevention. Our study thus contributes both to the understanding of viral pathogenesis and to an evidence‐based approach for dermatological care.

MC often spreads through direct contact with infected skin. However, whether contact with intact lesions can cause infection remains unclear. The MC virus only affects the surface of the body and does not spread internally [21]. Based on the direct observation of MC virus particles, our findings strongly suggest that the corneum layer serves as a strong barrier against MC virus skin penetration, which may explain phenomena such as the rarity of lesions on the palms of the hands or soles of the feet, where the corneum is thick [22, 23, 24], and why individuals with compromised skin barriers in conditions such as atopic dermatitis are more prone to developing MC [21, 24]. Avoiding scratching and covering scars are therefore important preventive measures.

In conclusion, this study presents the first application of NanoSuit‐CLEM for MC particle observation in FFPE sections, offering insights into the mechanisms by which the virus spreads. Our findings show that while MC virus aggregates in the stratum corneum, it does not penetrate intact skin layers, underscoring the importance of skin integrity for preventing viral entry.

## Author Contributions

**Hideya Kawasaki:** conceptualization, methodology, validation, data curation, formal analysis, resources, writing the original draft, and reviewing. **Yuri Sakano:** methodology, data curation, and reviewing. All authors have read and agreed to the published version of the manuscript.

## Ethics Statement

The Research Ethics Boards at the Hamamatsu University School of Medicine (approval number 18‐139) and Chutoen General Medical Center (No. 147) approved this study.

## Consent

Informed consent was obtained from all participants.

## Conflicts of Interest

The authors declare no conflicts of interest.

## Supporting information

Supplemental Figure 1.

## Data Availability

The data that support the findings of this study are available from the corresponding author upon reasonable request. No data are available for this study as no data sets were generated or analyzed during the current study.
