# Supplementary material for: Visualization of Molluscum Contagiosum Virus in FFPE Skin Sections Using NanoSuit‐CLEM: Ultrastructural Evidence of Viral Spread via Skin Barrier Disruption
Source: Immun Inflamm Dis. 2025 Jun 3;13(6):e70212. doi: 10.1002/iid3.70212 (PMC12131199; doi:10.1002/iid3.70212)
Supplement: Supplementary file 1 — Supplemental Figure 1. [file IID3-13-e70212-s001.docx]

Supplemental Figure 1


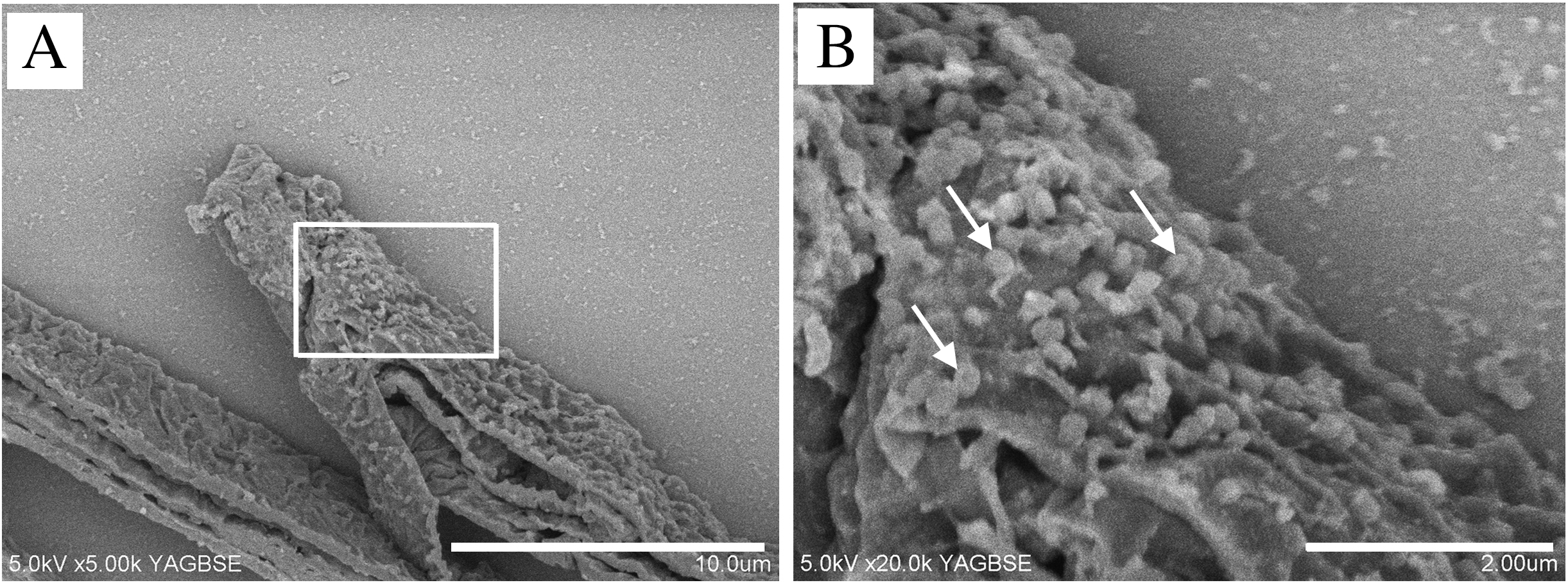


(**A**) FE-SEM images following Mayer's hematoxylin and lead staining. Viral particles exist on the corneum surface (BSE mode). White bar represents 10 μm. (**B**) Magnification of square region of A. White MC virus particles are clearly observed (white arrows) (BSE mode). Bar represents 2 μm.
